# Identification of prohormones and pituitary neuropeptides in the African cichlid, *Astatotilapia burtoni*

**DOI:** 10.1186/s12864-016-2914-9

**Published:** 2016-08-19

**Authors:** Caroline K. Hu, Bruce R. Southey, Elena V. Romanova, Karen P. Maruska, Jonathan V. Sweedler, Russell D. Fernald

**Affiliations:** 1 Department of Biology, Stanford University, Stanford, CA 94305 USA; 2 Department of Animal Sciences, University of Illinois at Urbana-Champaign, Urbana, IL 61801 USA; 3 Department of Chemistry and the Beckman Institute, University of Illinois at Urbana-Champaign, Urbana, IL 61801 USA; 4 Department of Biological Sciences, Louisiana State University, Baton Rouge, LA 70803 USA; 5 Present address: Department of Organismic and Evolutionary Biology, Harvard University, Cambridge, MA 02138 USA

**Keywords:** Prohormone, Neuropeptide, Cichlid, *Astatotilapia burtoni*, Mass spectrometry

## Abstract

**Background:**

Cichlid fishes have evolved remarkably diverse reproductive, social, and feeding behaviors. Cell-to-cell signaling molecules, notably neuropeptides and peptide hormones, are known to regulate these behaviors across vertebrates. This class of signaling molecules derives from prohormone genes that have undergone multiple duplications and losses in fishes. Whether and how subfunctionalization, neofunctionalization, or losses of neuropeptides and peptide hormones have contributed to fish behavioral diversity is largely unknown. Information on fish prohormones has been limited and is complicated by the whole genome duplication of the teleost ancestor. We combined bioinformatics, mass spectrometry-enabled peptidomics, and molecular techniques to identify the suite of neuropeptide prohormones and pituitary peptide products in *Astatotilapia burtoni*, a well-studied member of the diverse African cichlid clade.

**Results:**

Utilizing the *A. burtoni* genome, we identified 148 prohormone genes, with 21 identified as a single copy and 39 with at least 2 duplicated copies. Retention of prohormone duplicates was therefore 41 %, which is markedly above previous reports for the genome-wide average in teleosts. Beyond the expected whole genome duplication, differences between cichlids and mammals can be attributed to gene loss in tetrapods and additional duplication after divergence. Mass spectrometric analysis of the pituitary identified 620 unique peptide sequences that were matched to 120 unique proteins. Finally, we used *in situ* hybridization to localize the expression of galanin, a prohormone with exceptional sequence divergence in cichlids, as well as the expression of a proopiomelanocortin, prohormone that has undergone an additional duplication in some bony fish lineages.

**Conclusion:**

We characterized the *A. burtoni* prohormone complement. Two thirds of prohormone families contain duplications either from the teleost whole genome duplication or a more recent duplication. Our bioinformatic and mass spectrometric findings provide information on a major vertebrate clade that will further our understanding of the functional ramifications of these prohormone losses, duplications, and sequence changes across vertebrate evolution. In the context of the cichlid radiation, these findings will also facilitate the exploration of neuropeptide and peptide hormone function in behavioral diversity both within *A. burtoni* and across cichlid and other fish species.

**Electronic supplementary material:**

The online version of this article (doi:10.1186/s12864-016-2914-9) contains supplementary material, which is available to authorized users.

## Background

Ray-finned fishes comprise ~50 % of all vertebrate species and of these, teleost fishes are the most diverse clade. They are found in most aquatic habitats and exhibit vast behavioral differences between species. Among teleost fishes, cichlids are one of the most species-rich families and the African cichlids, in particular, provide exceptional and unique opportunities for understanding speciation and behavioral adaptations in the African Great Lakes [1]. Cichlid phenotypic diversity in behavior, body shape, coloration, and ecological specialization is unparalleled. Some substrates for cichlid morphological diversity include well-conserved morphogen systems, and potentially the Hox gene clusters [2–4]. Although cichlid behavioral diversity has encouraged behavioral and neurobiological studies directed at understanding how brain evolution has been shaped by natural and sexual selection, the molecular and cellular bases of teleost/cichlid behavioral diversity are still largely unknown. This status is poised to change. Not only have analyses shown remarkable social and cognitive skills associated with cichlid group living [5], the recent sequencing of five cichlid genomes [6] and the development of cichlid transgenesis techniques [7, 8] have opened the door to greater understanding of the underlying mechanisms.

It has been speculated that the rich diversity and complexity of behaviors found in teleosts partially derives from a whole genome duplication (WGD) in the teleost ancestor after the divergence from other vertebrate lineages [9, 10]. Approximately 85 % of genes resulting from this duplication were subsequently lost, but amongst the retained duplicates, genes associated with brain function are overrepresented [9, 11]. Retained duplicated genes can be a source of novel gene function as they frequently undergo subfunctionalization, neofunctionalization, or some combination of the two [12]. In the haplochromine cichlid lineage, for example, neofunctionalization of a paralog has been linked to the morphological innovation of male fin spots involved in mating behavior [13].

Neuropeptides play a pivotal role in both ancient and recently derived examples of animal behavior. For example, the galanin peptide is functionally associated with the regulation of feeding, anxiety-related behaviors, and parental behavior in mammals [14, 15]. Galanin’s orexigenic function has been described in teleosts, but whether it serves additional neuroendocrine or behavioral functions is unknown [16]. In *Astatotilapia burtoni*, whole brain galanin (*GAL*) expression is higher in socially dominant males compared to socially subordinate males [17]. Duplication of prohormone genes such as proopiomelanocortin (*POMC*) that encode multiple neuropeptides may similarly be a source of behavioral innovation through change in expression and sequence. Teleosts possess duplicate *POMC* genes and *A. burtoni*, as well as several other teleost lineages, have undergone a more recent *POMC1* duplication to generate *POMC1A* and *POMC1B* [18]. Many *POMC* peptide products, including melanocortins and *β*-endorphin, exert pleiotropic functions in multiple tissues, including the nervous system, reproductive system, and skin. Thus, regulation of *POMC* is a possible mechanistic link between behavior, physiology, and coloration within and across species [19]. Identification of which *POMC* versions are being expressed and which peptides are present is essential to understanding this link.

This effort to characterize the prohormone gene and novel neuropeptide complement for *A. burtoni* is the first comprehensive bioinformatic survey of any single ray-finned fish species. *A. burtoni* is a haplochromine cichlid with an advantageous phylogenetic position and a well-characterized natural history [20], and has undergone extensive physiological, neurobiological, and molecular analyses [21]. Molecular phylogenetics place this species in a sister group to the extremely large cichlid species flocks in Lakes Victoria and Malawi in East Africa. *A. burtoni* is hypothesized to be similar to the ancestor of these flocks because it is a trophic generalist endemic to the neighboring Lake Tanganyika and surrounding rivers [22, 23]. Thus, discoveries about the peptidome of *A. burtoni* are significant to the entire ‘modern haplochromine’ lineage, which represents ~7 % of all extant teleosts.

## Results and discussion

We surveyed the *A. burtoni* genome for prohormone genes as well as the major processing enzymes used to form bioactive peptides from the prohormone proteins. While previous studies have only examined individual prohormones and specific prohormone families, our study provides a comprehensive summary of all known prohormones. As the final bioactive complement requires the prohormone and appropriate processing enzymes, we also characterized the peptides themselves within the endocrine pituitary using mass spectrometry (MS). Finally, *in situ* hybridization was used to localize the expression of 2 prohormones, *GAL* and *POMC*.

Our survey identified 158 *A. burtoni* genes, with 148 prohormone genes and 10 prohormone convertase subtilisin/kexin (*PCSK*) genes. All prohormone genes, including current accession numbers and genomic locations [6], are provided in Additional file 1: Table S1. All predicted sequences can be found in FASTA format in Additional file 2: Text S1, which also includes 7 genes with 2 splice variants and 1 gene with 3 splice variants. All predictions except glucagon II (*GCG2*), kisspeptin-2 (*KISS2*), neuropeptide VF precursor (*NPVF*), prokineticin 2 (*PROK2*) and parathyroid hormone A (*PTHA*) were supported by *A. burtoni* expressed sequence tag (EST) data. Predictions without *A. burtoni* EST data were all supported by *Oreochromis niloticus* (Nile tilapia) EST data. Gastrin-releasing peptide (*GRP*) was not identified in the assembly but was identified from *A. burtoni* and *O. niloticus* EST data.

The 148 prohormone genes consisted of 6 genes that were only identified in fish, 21 genes identified with a single copy, and 39 genes with at least 2 duplicated copies (Table 1). Compared to the average rate of gene retention following the teleost WGD of 15 % [9, 11], we found prohormone genes to be retained at 41 % (39 genes with multiple copies out of 96 unique prohormone genes). Almost 66 % of the currently identified prohormone families contained at least one gene duplication (23 out of 35 families) (Table 1). The 6 predictions lacking direct mammalian counterparts are likely due to either gene loss in tetrapods or additional duplication after divergence of cichlid and mammals.

**Table 1:** Predicted *A. burtoni* prohormone families and duplication status

Neuropeptide Family	N G^a^	N D^b^	Gene Symbols^c^	References
Apelin	1	0	*APLN*	[70]
Augurin	1	0	*AUGN*	
AVIT/Prokineticin	2	0	*PROK1*; *PROK2*	
Bombesin/Neuromedin-B/Ranatensin	3	2	*GRP*; *NMB1*; *NMB2*	[113]
Calcitonin	8	4	*ADM1A*; *ADM1B*; *ADM2A*; *ADM2B*; *ADM5*; *CALCA*; *CALCB*; *IAPP*	[114]
CART	6	6	*CARTPT1*; *CARTPT2*; *CARTPT3*; *CARTPT4*; *CARTPT5*; *CARTPT6*	[71]
clpA/clpB family. Torsin subfamily.	1	0	*TOR2A*	
Corticotrophin	5	2	*CRH1A*; *CRH1B*; *UCN2*; *UCN3*; *UTS1*	[50, 115–117]
Endothelin/Sarafotoxin	6	6	*EDN1A*; *EDN1B*; *EDN2A*; *EDN2B*; *EDN3A*; *EDN3B*	[118]
Gastrin/Cholecystokinin	3	2	*CCK1*; *CCK2*; *GAST*	[119]
Ghrelin/Motilin-related	2	0	*GHRL*; *MLN*	[119, 120]
Glucagon	8	4	*ADCYAP1A*; *ADCYAP1B*; *GCG1A*; *GCG1B*; *GCG2*; *GHRH*; *GIP*; *VIP*	[35, 37, 121]
Gonadotropin-releasing hormone	3	0	*GNRH1*; *GNRH2*; *GNRH3* ^*d*^	[28, 122–124]
Granin	9	4	*CHGA*; *CHGB*; *PCSK1N*; *SCG2A*; *SCG2B*; *SCG3*; *SCG5*; *VGF1*; *VGF2*	[73, 125, 126]
Hepcidin	5	5	*HAMP1*; *HAMP2*; *HAMP3*; *HAMP4*; *HAMP5*	[68]
Insulin	5	2	*IGF1*; *IGF2*; *IGF3* ^*d*^; *INS1*; *INS2*	[32, 127]
Kisspeptin/Galanin/Spexin	4	2	*KISS2* ^*d*^; *GAL*; *SPX1*; *SPX2*	[61, 62, 128–130]
Melanin-concentrating hormone	2	2	*PMCH1*; *PMCH2*	[131]
Natriuretic peptide	6	4	*NPPA*; *NPPB*; *NPPC1*; *NPPC2*; *NPPC3*; *NPPC4*	[70]
Neuropeptide B/W	2	2	*NPB1*; *NPB2*	[53]
Neurotensin	1	0	*NTS*	
Neuromedin	2	0	*NMS*; *NMU*	[132]
Neuropeptide Y	4	4	*NPY1*; *NPY2*; *PYY1*; *PYY2*	[54, 55]
Nucleobindin	3	2	*NUCB1*; *NUCB2A*; *NUCB2B*	
Opioid	7	5	*PDYN*; *PENK*; *PNOC1*; *PNOC2*; *POMC1A*; *POMC1B*; *POMC2*	[46–48, 104, 133–135]
Orexin	1	0	*HCRT*	[136]
Osteocrin	1	0	*OSTN*	
Oxytocin/Vasopressin	2	0	*AVP*; *OXT*;	[137]
Parathyroid	6	4	*PTH1A*; *PTH1B*; *PTH2*; *PTHLH1*; *PTHLH2*; *PTHLH3* ^*d*^	[57, 58, 138, 139]
PDGF/VEGF growth factor	13	10	*FIGF*; *PDGFA1*; *PDGFA2*; *PDGFB1*; *PDGFB2*; *PDGFC*; *PDGFD*; *PGF1*; *PGF2*; *VEGFA1*; *VEGFA2*; *VEGFC1*; *VEGFC2*	[33, 34]
Relaxin	6	4	*INSL3*; *INSL5A*; *INSL5B*; *RLN1*; *RLN3A*; *RLN3B*	[33, 34, 140]
Rfamide peptide	5	2	*NPFF*; *NPVF*; *QRFP*; *PRLH1*; *PRLH2*	[141–143]
Somatostatin/Urotensin	8	4	*SST1*; *SST2*; *SST3*; *SST5*; *URP1* ^*d*^; *URP2* ^*d*^; *UTS2A*; *UTS2B*	[39, 40]
Tachykinin	6	6	*TAC1A*; *TAC1B*; *TAC3A*; *TAC3B*; *TAC4A*; *TAC4B*	[144]
Thyrotropin-releasing hormone	1	0	*TRH*	[145]

^a^N G: Number of identified gene sequences within family

^b^N D: Number of identified gene sequences within family that have been duplicated

^c^Gene Symbol: Based on *Homo sapiens* gene symbols where probable duplicated genes are denoted with either a number or lower case letter. Full gene names and symbols are provided in Additional file 2

^d^Prohormones lacking direct mammalian counterparts

### Gonadotropin-releasing hormone and oxytocin/vasopressin families

The gonadotropin-releasing hormone (*GNRH*) and oxytocin/vasopressin families comprise the most functionally well-characterized prohormones in *A. burtoni* [24, 25]. Our genomic survey confirms previous findings of single copies of three *GNRH* genes (*GNRH1*, *GNRH2*, and *GNRH3*), as well as single copies of oxytocin (*OXT*) and arginine vasopressin (*AVP*). In *Oryzias latipes* (Japanese medaka fish), GnRH3 peptide is produced in the terminal nerve ganglion and modulates social behavior [26]. It is unknown whether this neuromodulatory role is shared across teleosts and whether this role is fulfilled by GnRH1 in tetrapods, which lack *GNRH3* [27, 28]. Evidence based on the receptors suggests that *GNRH*, *OXT*, *AVP* and neuropeptide S (*NPS*) genes share a common ancestor [29, 30]. Analysis of *Branchiostoma floridae* (lancelet) *AVP*, *GNRH*, and *NPS* prohormones indicate conserved synteny of these prohormones [31]. It is theorized that the *NPS* system was lost in ray-finned fish after the duplication of an ancestral system that resulted in the OXT/AVP system and the *NPS* system [30]. Consistent with this hypothesis, there was no evidence of *NPS* in *A. burtoni*.

### Insulin and relaxin families

Duplicate copies of insulin (*INS1* and *INS2*) and single copies of insulin-like growth factors 1 (*IGF1*), 2 (*IGF2*), and 3 (*IGF3*) were identified. *IGF3* is a teleost-specific, gonad-specific prohormone [32]. Following Wilkinson et al. [33] and Yegorov and Good [34], 2 copies of relaxin 3 (*RLN3A* and *RLN3B*) and insulin-like 5 (*INSL5A* and *INSL5B*) and single copies of relaxin 1 (*RLN1*) and insulin-like 3 (*INSL3*) were also identified. Multiple sequence alignment indicated that *INS1*, *INS2*, *IGF1*, and *IGF2* were more similar to the mammalian counterparts than other members of the relaxin family. Similar to Yegorov and Good [34], only the 2 copies of *A. burtoni RLN3* showed more similarity to the *Homo sapiens* (human) relaxin family counterparts than the other identified genes. The other 3 relaxin members had intermediate similarity between the *H. sapiens RLN3* and the other *H. sapiens* relaxin family members.

### Glucagon family

Searching the *A. burtoni* genome identified 6 of the 7 known members of the glucagon family: 2 glucagon 1 (*GCG1*) copies (*GCG1A* and *GCG1B*), 2 adenylate cyclase activating polypeptide 1 copies (*ADCYAP1A* and *ADCYAP1B*), and single copies of glucagon 2 (*GCG2*), gastric inhibitory polypeptide (*GIP*), growth hormone releasing hormone (*GHRH*), and vasoactive intestinal peptide (*VIP*). All identified prohormones were more similar in sequence to their respective *H. sapiens* and *Gallus gallus* (chicken) homologues than the other glucagon members. *GCG2* is similar to glucagon type II found in *O. latipes*, *G. gallus*, and *Xenopus (Silurana) tropicalis* (western clawed frog) [35, 36]. This second glucagon has been lost in mammals since there are no detectable sequences or conserved synteny found in mammalian genomes [35]. No secretin (*SCT*) was identified in *A. burtoni*, as it is considered lost in teleosts, but the *SCT* receptor has been identified [37, 38].

### Somatostatin and urotensin II families

Following Tostivint et al. [39, 40], single copies of 4 members of the somatostatin (*SST*) family were identified (*SST1*, *SST2*, *SST3*, and *SST5*) in *A. burtoni*, but no evidence of other *SST* versions. It is proposed that the somatostatin and urotensin II families are related by an early evolutionary event [39–43]. The urotensin II family consists of 2 members, urotensin 2 (*UTS2*) and urotensin 2B (*UTS2B*), that are widespread through many taxa including invertebrates [44]. In addition, the urotensin II family consists of two additional members, urotensin II-related peptide 1 (*URP1*) and urotensin II-related peptide 2 (*URP2*), which appear to be absent in tetrapods [40]. Both *A. burtoni URP1* and *URP2* contain the urotensin II domain and the dibasic cleavage site necessary to produce the urotensin 2B neuropeptide. Injection of URP1 and URP2 peptides in *Oncorhynchus mykiss* (rainbow trout) found that these peptides had similar, but not identical, effects on locomotor behavior and cardio-respiratory physiology to UTS2, suggesting some subfunctionalization within this family in ray-finned fishes [45].

### Opioid peptide prohormone genes

Duplicates of prepronociceptin (*PNOC*; *PNOC1* and *PNOC2*), and single copies of proenkephalin (*PENK*) and prodynorphin (*PDYN*), were identified in *A. burtoni*. Similar to the *Verasper moseri* (barfin flounder) and *O. mykiss* [46], 3 versions of *POMC* (*POMC1A*, *POMC1B*, and *POMC2*) were also identified.

It is hypothesized that these opioid genes are related through two rounds of genomic duplication [47, 48]. All 3 *POMC* sequences lacked the melanocyte-stimulating hormone (MSH) peptide, γ-MSH, consistent with the loss of γ-MSH in ray-finned fishes [48]. Only 2 *POMC* versions were similar across species, suggesting these are duplicated copies from the teleost duplication and independent duplication events led to a second paralog.

Comparisons of *POMC* sequences have indicated that *POMC1A* and *POMC1B* are a result of a tandem duplication in the teleost lineage near when the Pleuronectiformes (e.g., flounders) split from Perciformes (e.g., cichlids) [18]. Further, Harris et al. [18] observed a tandem duplication within the *POMC2* gene that encompassed part of the N-terminal fragment, all of α-MSH, and part of the adrenocorticotropic hormone that arose before the radiation of cichlids but sometime after the radiation of Labridea (wrasses). A novel ε-MSH peptide was proposed [18], but this peptide is unlikely to occur or be bioactive in *A. burtoni*. While the proposed cleavage fits the RxxK motif that is cleaved as part of the known motif model [49], the sequence lacks a suitable amidation site that is present in all MSH peptides.

### Corticotropin family

Duplicated copies of corticotropin-releasing hormone 1 (*CRH1A* and *CRH1B*), and single copies of urotensin 1 (*UTS1*), urocortin 2 (*UCN2*), and urocortin 3 (*UCN3*) genes were identified. Alignment to the mammalian versions indicated *A. burtoni CRH*, *UTS1*, and *UCN3* were similar to mammalian corticotropin-releasing hormone (*CRH*), urocortin 1 (*UCN1*), and *UCN3*, respectively. Similar to Boorse et al. [50], *UCN2* was intermediate between mammalian *UCN2* and *UCN3* with only the *UCN2* domain shared. It is proposed that a genome duplication prior to the divergence of actinopterygian and sarcopterygian fishes gave rise to duplicated *UCN3* and *CRH* genes [50, 51]. In the case of *CRH*, one gene duplicate, *CRH2*, was lost in teleost fishes and eutherian mammals, and *A. burtoniCRH1A* and *CRH1B* are likely duplicates of an ancestral CRH1 [51, 52].

### Neuropeptide B/W family

We found duplicate versions of neuropeptide B (*NPB*), *NPB1* and *NPB2*, but not neuropeptide W (*NPW*) in *A. burtoni*. Both the prohormones and neuropeptide receptors are highly related [53]. Both *NPB* and *NPW* have been identified in the genome of *Monodelphis domestica* (opossum; *NPB*: [GenBank:XM_001379652.2]; *NPW*: [GenBank:XM_007499923.1]) and *Xenopus laevis* (African clawed frog; *NPB*: [GenBank:XM_002937305.3]; *NPW*: [GenBank:XM_004918054.2]). Although *NPW* was not identified in the avian genomes of *G. gallus* and *Taeniopygia guttata* (zebra finch), an *NPW-like* sequence is identified in *Pseudopodoces humilis* (ground tit; [GenBank:XM_005523213.1]). Homology searching in *Latimeria chalumnae* (coelacanth) also indicated both *NPB* ([GenBank:XM_005989108.1]) and *NPW* (match on scaffold01390 NCBI Reference Sequence: [GenBank:NW_005820400.1]). Thus, our findings in *A. burtoni* support that *NPW* either arose after the split of the teleosts from other vertebrates or was lost in the teleost lineage.

### Neuropeptide Y family

The neuropeptide Y family consists of neuropeptide Y (*NPY*), pancreatic prohormone (*PPY*), and peptide tyrosine-tyrosine (*PYY*) that arose by gene duplication [54, 55]. Two copies of NPY (*NPY1* and *NPY2*) and two of PYY (*PYY1* and *PYY2*) were identified in *A. burtoni*, as well as the ray-finned fish-specific pancreatic peptide Y, which has been recognized as a duplicate of *PYY* [54]. *PPY* was not identified, consistent with a duplication event after tetrapod divergence [55]. The *L. chalumnae* sequence ([GenBank:XM_005992227.1]) containing the partial pancreatic polypeptide sequence reported by Larhammar and Bergqvist [56] was more similar to *PYY* than the other neuropeptide Y family members.

### Parathyroid hormone family

The parathyroid family consists of parathyroid hormone 1 (*PTH1*), parathyroid hormone 2 (*PTH2*) and parathyroid hormone-related hormone (*PTHLH*) [57, 58]. Duplicate versions of *PTH1* (*PTH1A* and *PTH1B*) and *PTHLH* (*PTHLH1* and *PTHLH2*) were identified, and a single version *PTH2* were identified. In addition, a third *PTHLH* gene (*PTHLH3*) similar to the *Danio rerio* (zebrafish) predicted gene ([GenBank:XM_005168285.2]) was identified that had intermediate homology between the *PTH* and *PTHLH* genes and appears to have been lost in eutherian mammals [57, 58].

### RFamide and kisspeptin/galanin/spexin families

The RFamide family consists of prohormones that produce C-terminal arginine and amidated phenylalanine bioactive peptide [59, 60]. It has been proposed that kisspeptin (*KISS*), *GAL* and spexin (*SPXN*) belong to the same family due to the sequence similarly of prohormones and receptors and that *SPXN* can activate the *GAL* receptors [61]. The relationship between these families is undergoing further revision as it has been suggested that *KISS* and prolactin-releasing hormone (*PRLH*) may not belong in the RFamide family [62].

Duplicate copies of *SPXN* (*SXPN1* and *SPXN2*) and *PRLH* (*PRLH1* and *PRLH2*), and single copies of neuropeptide FF-amide peptide precursor (*NPFF*); *NPVF*; pyroglutamylated RFamide peptide (*QRFP*), *GAL* and *KISS2* were identified. There was no evidence for an *A. burtoni* galanin-like peptide (*GALP*) or kisspeptin-1 (*KISS1*).

The presence of *KISS2* and the lack of *KISS1* in *A. burtoni* is consistent with *KISS* gene evolution [63]. Interestingly, some ray-finned fish and *Ornithorhynchus anatinus* (platypus) have both *KISS1* and *KISS2*, whereas eutherian mammals have maintained only *KISS1*. There is some indication of a *KISS2-like* gene in apes [64] that may be a pseudogene because it is a single exon compared to the 2 exons in other species. The presence of a single *GAL* gene appears to be consistent with other teleost species, with the exception of Cypriniformes (e.g., *D.rerio* and *Carassius auratus* (goldfish)) that appear to have a duplication of *GAL* (*GAL1* and *GAL2*) [16, 65]. Two *GAL* isoforms were determined from EST evidence. The longer of the *A. burtoni* splice isoforms introduces an in-frame insertion of 72 bp due to alternative splicing of exon 3 and 4. A similarly generated splice isoform has also been identified in other teleosts and avian species [65, 66].

The lack of evidence of *GALP* is consistent with our previous studies suggesting it may only be present in some eutherian mammals [67]. The partial *L. chalumnae GALP*, predicted by Kim et al. [61], appears to be a duplicated *GAL* gene. Homology indicated that it is more similar to the *X. laevis GAL2* ([GenBank:XM_004916293.1]) than the *X. laevis GAL1* ([Genbank:XM_002941642.3]). It is likely that this is a duplicated *GAL* gene rather than *GALP* because the *X. laevis* sequence contains the galanin message associated peptide (GMAP), which is not present in mammalian *GALP*, and because *X. laevis* and *L. chalumnae GAL2* genes lack synteny with *H. sapiens* GALP [61].

### Hepcidin antimicrobial peptide

Five *HAMP* sequences were identified in *A. burtoni* (*HAMP1*, *HAMP2*, *HAMP3*, *HAMP4* and *HAMP5*), however 2 sequences (*HAMP2* and *HAMP4*) were virtually identical. These 2 sequences were located on different contigs and the location of one sequence at the end of a contig indicates these may be the result of an assembly error. The different *HAMP* versions are a consequence of WGD and additional tandem gene duplication, possibly related to host-pathogen interaction or changes in oxygen availability [68, 69].

### Natriuretic peptides

Single copies of natriuretic peptide A (*NPPA*) and natriuretic peptide B (*NPPB*) were identified as well as the expected 4 copies of natriuretic peptide C (*NPPC1*, *NPPC2*, *NPPC3*, and *NPPC4*) [70]. Multiple chromosomal duplications resulted in 4 versions of the ancestral *NPPC* gene with the subsequent loss of the *NPPC1*, *NPPC2* and *NPPC3* versions in tetrapods. Prior to the *NPPC3* loss in tetrapods, tandem duplication of the *NPPC3* gene gave rise to *NPPA* and *NPPB* [70], which is evident by less than 6,000 bps between these genes in the *A. burtoni* assembly.

### CART prepropeptide

Similar to *O. latipes*, 6 different CART prepropeptide (*CARTPT*) prohormones were identified in *A. burtoni. CARTPT1* was most similar to mammalian *CARTPT* than the other *CARTPT* prohormones identified. The relationship between different prohormones is unclear because all versions are located on different scaffolds in the current *A. burtoni* genome assembly. Multiple sequence alignment suggests greater sequence similarity between two pairs of *CARTPT* prohomones: *CARTPT1* with *CARTPT2*, and *CARTPT3* with *CARTPT4. O. latipes* also has 6 *CARTPT* copies [71] and *D. rerio* has 4 *CARTPT* copies [72].

### The granin family

*A. burtoni* orthologs were identified for the mammalian granin family members chromogranin A (*CHGA*), chromogranin B (*CHGB*), secretogranin II (*SCG2*), secretogranin III (*SCG3*), secretogranin V (*SCG5*), proprotein convertase subtilisin/kexin type 1 inhibitor (*PCSK1N*), and vascular endothelial growth factor (*VGF*) [73]. In addition, two copies of *SCG2* (*SCG2A* and *SCG2B*) and *VGF* (*VGF1* and *VGF2*) were found. Although a match for GNAS complex locus was found, there was no homology to the neuroendocrine secretory protein-55 isoform. Similar to Kudo et al. [74], there was limited similarity between mammalian and the identified versions of *PCSK1N* genes; the *A. burtoni* version contained PEN-like and little-LEN peptides but lacked the SAAS peptides. This suggests that the gain of *PCSK1N* functionality is only in mammals.

### Salusin peptides and torsin family 2, member A

Unlike most prohormones, the *TOR2A* gene undergoes alternative splicing where one isoform is cleaved into the salusin peptides [75]. While the *A. burtoni TOR2A* gene was identified, there was no predicted isoform that could produce the salusin peptides. Subsequent searches for the salusin peptides in other species indicate that this isoform may have only arisen within eutherian mammals.

### Prohormone convertases family

The bioactive peptides produced from the prohormones described above depend on the specific set of prohormone convertases (as well as other enzymes responsible for post-translational modifications) present [76]. A search of the *A. burtoni* genome identified *PCSK* type 1 (*PCSK1*), 2 (*PCSK2*), 5 (*PCSK5*), 7 (*PCSK7*), and 9 (*PCSK9*) genes. There were no matches to mammalian *PCSK* type 4 and 6 genes. Generally these *PCSK* genes showed higher similarity to their mammalian counterparts than to each other. Two copies of *PCSK5* (*PCSK5A* and *PCSK5B*) and *FURIN* (*FURIN1* and *FURIN2*) were identified and single copies of *PCSK1*, *PCSK2*, *PCSK7*, *PCSK9*, and membrane-bound transcription factor peptidase, site 1 (*MBTPS1*) were identified. Only the *PCSK5B* gene was similar to the mammalian version, with *PCSK5A* appearing to be a paralog of the mammalian gene. An identified *PCSK-like* gene may be an incomplete gene or an assembly error because the prediction lacks the FU domains that are present in other *PCSK* genes.

### Tandem mass spectrometry peptide identification in the pituitary

**Fig. 1:**
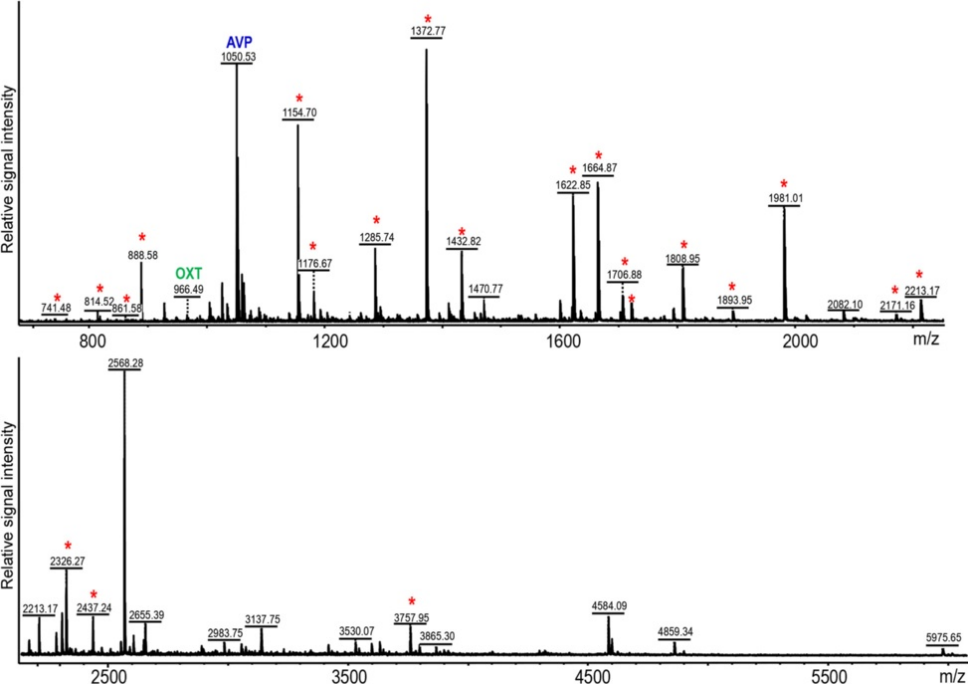
Peptide mass fingerprinting of *POMC1A* in male *Astatotilapia burtoni* pituitary by direct tissue MALDI-TOF MS. Masses matching the *POMC1A* peptides subsequently confirmed by tandem MS in the pituitary extracts are labeled with asterisks; masses matching oxytocin and arginine vasopressin are labeled by gene symbols *OXT* and *AVP*, respectively. List of all mass matches to sequenced peptides is given in Additional file 3: Table S2

Peptides resulting from the previously detected prohormones were identified using MS-based peptidomics [77–79]. We first assessed the feasibility of peptidomic experiments by testing peptide content in freshly frozen individual pituitaries using matrix-assisted laser desorption/ionization (MALDI) time-of-flight (TOF) MS. Mass fingerprinting of peptides measured by direct tissue MALDI-TOF MS detected and putatively identified 46 peptides from 12 proteins, including 29 peptides from *POMC1A* (Fig. 1), and all peptides were subsequently confirmed by tandem MS (Additional file 3: Table S2).

Tandem MS detected a total of 680 peptides from 620 unique peptide sequences after accounting for different modifications on the same sequence (Additional file 4: Table S3). There were 22 peptides detected with a single amino acid substitution compared to the predicted sequence from the genome, which may be due to genetic differences between the *A. burtoni* populations used for tandem MS and genome sequencing. The unique peptide sequences were matched to 125 unique proteins. Proteins identified by more than 9 unique characterized peptides each are listed in Table 2.

**Table 2:** Pituitary proteins confirmed by the highest number of peptides using tandem MS

Type	Symbol	N. peptides	Gene locus	Accession No.	Gene name
Prohormone	*AVP*	16	LOC102310610	NP_001273257.1	arginine vasopressin
Prohormone	*CHGB*	9	LOC102304831	XP_005917770.1	chromogranin B
Prohormone	*GNRH1*	9	LOC102291227	NP_001273225.1	gonadotropin-releasing hormone 1
Prohormone	*NUCB2B*	9	LOC102313482	XP_005931689.1	nucleobindin 2B
Prohormone	*OXT*	26	LOC102312886	XP_005919106.1	oxytocin
Prohormone	*PCSK1N*	11	LOC102305204	XP_005931188.1	proprotein convertase subtilisin/kexin type 1 inhibitor
Prohormone	*PMCH1*	24	LOC102310003	XP_005921781.1	pro-melanin-concentrating hormone 1
Prohormone	*PMCH2*	14	LOC102314388	XP_005934290.1	pro-melanin-concentrating hormone 2
Prohormone	*POMC1A*	164	LOC102296817	NP_001273262.1	proopiomelanocortin 1A
Prohormone	*SCG2A*	13	LOC102306907	XP_005948590.1	secretogranin IIa
Prohormone	*SCG2B*	12	LOC102295815	XP_005938347.1	secretogranin IIb
Prohormone	*SCG3*	22	LOC102313744	XP_005949052.1	secretogranin III
Prohormone	*TAC1B*	10	none	none	tachykinin precursor 1B
Other	*ACTB*	13	LOC102293972	XP_005952745.1	actin, cytoplasmic 1-like isoform X1
Other	*CALR*	12	LOC102296215	XP_005936722.1	calreticulin-like
Other	*HBA1*	21	LOC102301295	XP_005920277.1	hemoglobin subunit alpha-A-like
Other	*HBB*	13	LOC102301591	XP_005920278.1	hemoglobin subunit beta-A-like
Other	*HSP90B1*	15	LOC102308602	XP_005929149.1	endoplasmin-like
Other	*PDIA3*	20	LOC102293196	XP_005931703.1	protein disulfide-isomerase A3-like
Other	*PDIA4*	9	LOC102309651	XP_005912150.1	protein disulfide-isomerase A4-like
Other	*PPIB*	9	LOC102303154	XP_005931742.1	peptidyl-prolyl *cis*-*trans* isomerase B-like

Most of the peptides detected (164) were derived from *POMC* prohormones. There were 42 peptides with at least one post-translational modification. No *α-MSH* was detected but an N-terminal peptide of *POMC1A* was detected, which was expected from the sequence [48]. Subsequent alignment of all *POMC* peptides indicated that most of these were derived only from *POMC1A* (120), 39 peptides were derived from an identical peptide region in *POMC1A* and *POMC1B*, and 7 peptides with an identical sequence in *POMC1A*, *POMC1B*, and *POMC2*. There were no peptides uniquely identified to *POMC1B* and *POMC2*, indicating that *POMC1B* and *POMC2* prohormones were not present at detectable levels.

Both pro-melanin-concentrating hormone 1 (*PMCH1*) and pro-melanin-concentrating hormone 2 (*PMCH2*) were detected with 24 and 14 peptides, respectively. In addition, a single peptide was detected that shared a sequence between 2 copies of melanin-concentrating hormone (*MCH*), *MCH1* and *MCH2*. This peptide corresponds to the *MCH* encoding sequence at the C-terminal of both *MCH* prohormones. Peptides from *PMCH1* corresponded to the same region as mammalian neuropeptide-glycine-glutamic acid. The predicted *PMCH2* sequence could not form this neuropeptide-glycine-glutamic acid peptide, and, unlike the mammalian prohormone sequence, this *PMCH1* peptide is surrounded by dibasic amino acids. Neither *PMCH1* nor *PMCH2* prediction included a peptide corresponding to neuropeptide-glutamic acid-isoleucine.

Both arginine vasopressin and oxytocin neuropeptides were detected as well as other peptides from their respective prohormones. Overall, *OXT* and *AVP* provided 26 and 16 unique peptides, respectively. Oxytocin and arginine vasopressin peptides were detected, as well as C-terminal peptides corresponding with copeptin from both *OXT* and *AVP*. The remaining peptides detected were *OXT* fragments from the neurophysin 1 peptide. The detection of these peptides and the similarity of prohormone sequences indicate that mammalian *OXT* has undergone greater divergence than *AVP* since the tetrapod divergence.

The other prohormone with multiple peptides was *SCG3*, in which the majority of the peptides were near the signal peptide or near the C-terminus. The peptides near the signal peptide likely correspond to polypeptides resulting from cleavage at the arginine pair following removal of the signal peptide. Peptides from this region have also been detected in mammals (e.g., Fricker et al. [80]). However, since neither region had obvious NeuroPred-predicted [81] cleavage sites, it is unclear whether these are post-processing degradation products of the large SCG3 peptide, or resulted from post-translational enzymatic cleavage.

**Fig. 2:**
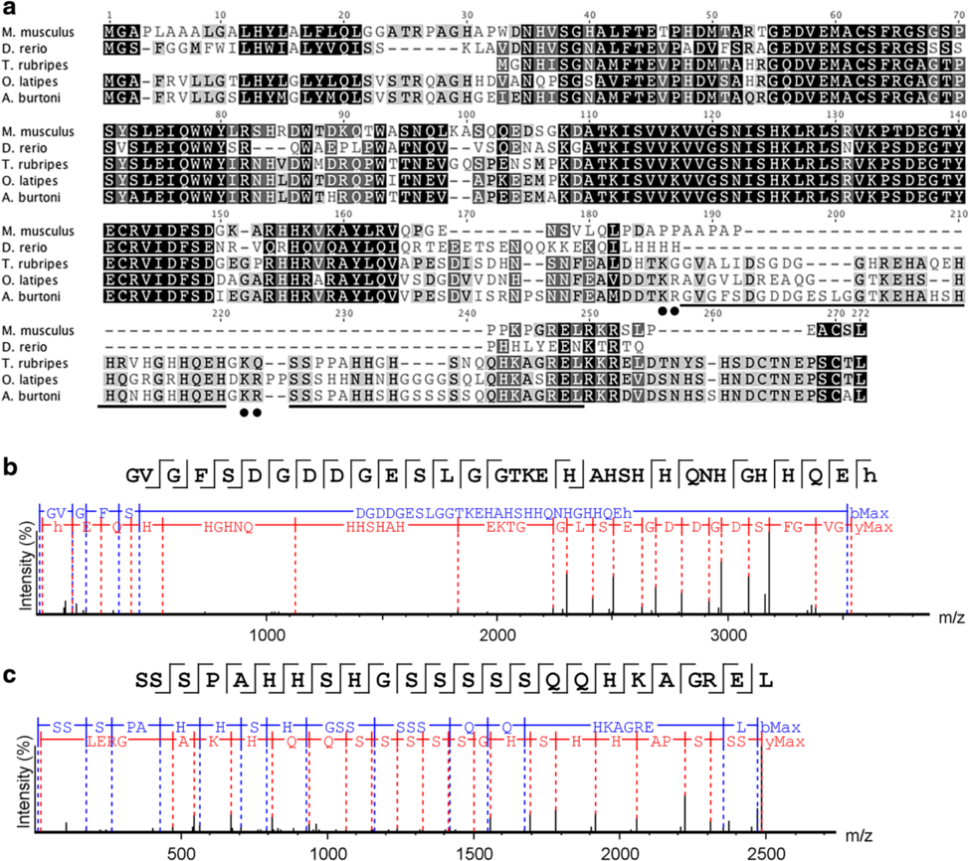
Identification of novel V-set and transmembrane domain-containing protein 2-like protein-like (*VSTM2A*) peptides. **a** Multiple sequence alignment *VSTM2A* proteins. C-terminal peptides identified with tandem MS are underlined and dibasic cleavage residues marked with black circles. Sequence accession numbers are *Mus musculus* (NP_941029.2), *Danio rerio* (XP_009300365.1), *Takifugu rubripes* (XP_011620087.1), and *Oryzias latipes* (XP_004070308.1). Fragment ion series for the 2 C-terminal peptides are shown in **b** and **c**

Examination of the peptides from V-set and transmembrane domain-containing protein 2-like protein-like (*VSTM2A*) indicates a novel C-terminal peptide (Fig. 2). Evaluation of the predicted protein sequence identified an immunoglobulin domain and dibasic sites commonly cleaved by prohormone convertases, and a signal peptide was predicted using SignalP v4.1 [82]. This protein is longer than the mammalian *VSTM2A* sequence. The observed peptides corresponded to regions between cleavage sites predicted by NeuroPred [81], with both vertebrate and invertebrate models. This predicted gene was found with similar length and dibasic sites in some ray-finned fish species (e.g., *O. latipes*: [GenBank:XM_004070260.2]; *Poecilia reticulata* (Guppy): [GenBank:XM_008413143.1]; *Cynoglossus semilaevis* (tongue sole): [GenBank:XM_008318238.1]; *Xiphophorus maculatus* (southern platyfish): [GenBank:XM_005807649.1]). However, some species, such as *Takifugu rubripes* (Japanese puffer) and *Lepisosteus oculatus* (spotted gar), appear to lack one or more of the dibasic sites, and others such as *L. chalumnae* and *D. rerio* lack any match to the region containing these peptides.

**Fig. 3:**
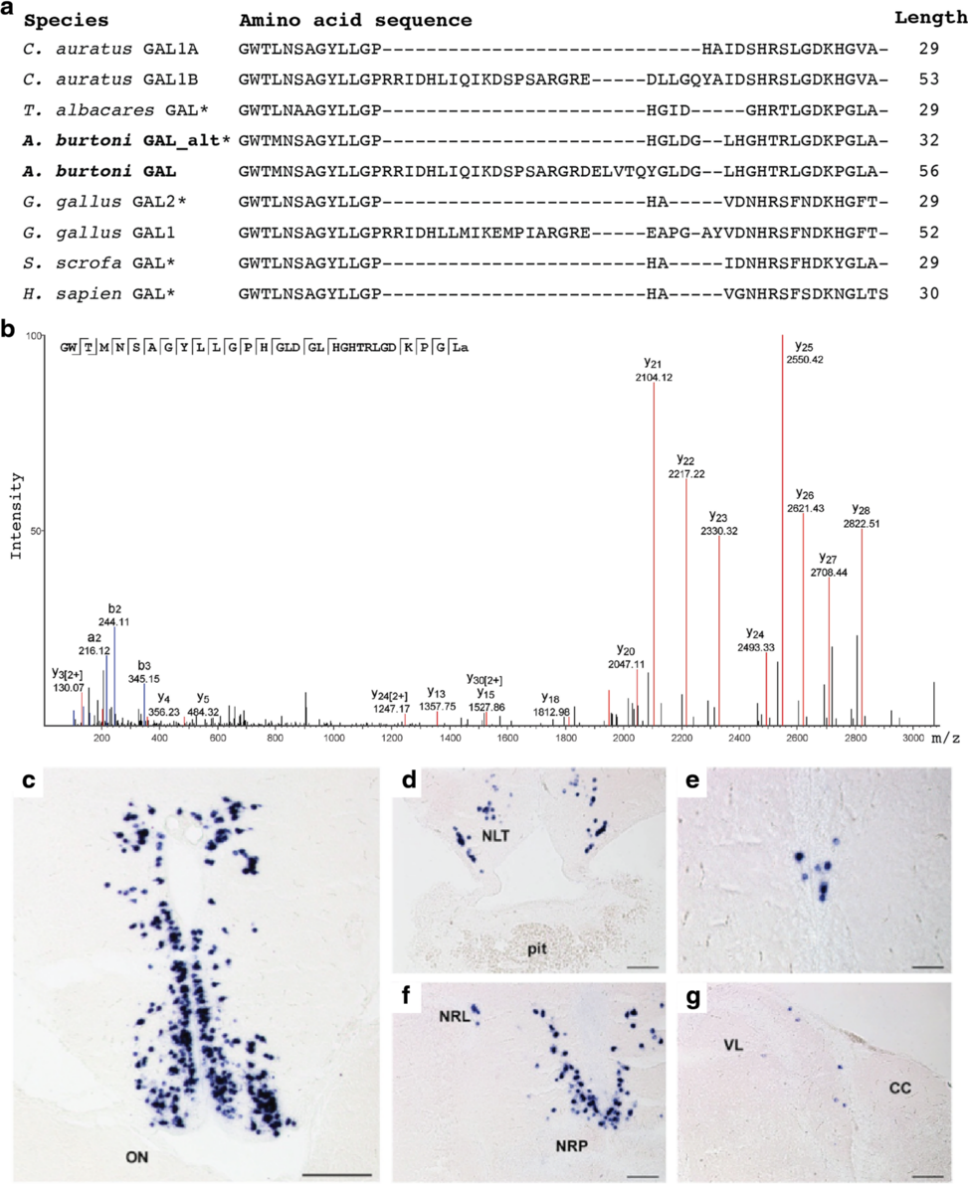
Characterization of galanin mature peptide and *GAL* prohormone expression. **a** Alignment of vertebrate galanin peptides. *Astatotilapia burtoni* sequences have a bolded species label. Sequences supported by peptide sequencing are marked with asterisks (*). Alignment made with the ClustalW program. **b** The *A. burtoni* galanin peptide fragmented and sequenced via tandem MS. **c**–**g** Representative transverse sections showing GAL-expressing neurons in the brain and pituitary. *GAL*-expressing cells were found in the preoptic area **c**, nucleus of the lateral tuberalis (NLT) **d**, medial region of the nucleus of the lateral recess (NRL) and in the region dorsal to the nucleus of the posterior recess (NRP) **e**, region of the periventricular nucleus of the posterior tuberculum **f**, and in the hindbrain along the lateral border of the vagal lobe (VL) **e**. CC, cerebellar crest; ON, optic nerve; pit, pituitary. Scale bars = 100 μm **c**; 25 μm **d**–**g**

Tandem MS identified peptides from alternatively spliced forms of *CHGB* and *GAL*. The *CHGB* isoforms are identical except that the longer *CHGB* isoform retains an intron that contains an MS-identified peptide. The galanin peptide detected by MS is encoded by the shorter *A. burtoni* splice isoform, and this peptide is distinguished by two aspects. First, the N-terminal 14 amino acids of galanin are highly conserved across vertebrates and are important for receptor binding [14]. Previously, the only known exception was in *Thunnus albacares* (yellowfin tuna), which has a serine to alanine substitution at position 6 [83]. *A. burtoni* galanin is also an exception, with a leucine to methionine substitution at position 4 (Fig. 3a). Second, the *A. burtoni* peptide is 32 amino acids long, making it the longest sequenced galanin peptide (Fig. 3b). The length of galanin is highly conserved across vertebrate evolution at 29 amino acids and previously only *H. sapiens* galanin was known to exceed this length [84]. Our understanding of galanin receptor function in teleosts is only beginning to emerge [85], therefore, the significance of this substitution is unknown. If *A. burtoni* galanin receptors are similar to *D. rerio* galanin receptors, and display affinity for galanin and spexin peptides, then the unusual form of *A. burtoni* galanin may suggest that there has been pronounced ligand-receptor co-evolution in the GAL/SPXN system in cichlids.

Detection of numerous peptides from non-secreted proteins such as hemoglobin subunit-α-like and β-like, endoplasmin-like, actin, and cytoplasmic 1-like protein, among others in the pituitary, is not surprising due to their ubiquitous nature. High protein sequence coverage was obtained for some of these proteins due to multiple detected peptides (Additional file 3: Table S2). In fact, enzymatic processing of cytosolic proteins can generate non-classical peptides that may have biological activity [86, 87]. In particular, hemoglobin-derived peptides, such as hemorphins and hemopressins, have diverse functions in various tissues and are expressed by neurons [87, 88]. Therefore, *A. burtoni* peptides from hemoglobin subunit-α-like (20 unique peptide sequences) and hemoglobin subunit-β-like (8 peptides) may in part represent bioactive peptides. The disulfide-isomerase proteins, protein disulfide-isomerase A3-like (18 unique peptide sequences), and protein disulfide-isomerase A4-like (9 peptides), regulate folding and redox state of proteins via formation, reduction, or isomerization of disulfide bonds [89]. Endoplasmin (15 unique peptides) is a molecular chaperone involved with the processing and transport of secreted proteins [90]. Actin, cytoplasmic 1-like (12 peptides), possibly reflects the role of actin in vesicle transport [91]. Calreticulin (12 peptides) is involved with maintaining adequate calcium levels in the system, and functions as a chaperone in the folding of other proteins [92].

### Localization of galanin prohormone expression

Galanin is one of the better characterized peptides within ray-finned fishes; having identified the mature *A. burtoni* galanin peptide, we next sought to identify G*AL*-expressing cells in the *A. burtoni* brain using *in situ* hybridization (Fig. 3c–g). The distribution of galanin in many ray-finned fish species has been investigated primarily through detection of galanin-like immunoreactivity using anti-porcine galanin antibodies [16]. These studies have shown that in ray-finned fish, pituitary galanin is exclusively neural in origin, rather than both neural and pituitary-derived, as seen in mammals [14]. The *Xiphophorus* and *Anableps* genera may be exceptions [93, 94]. *In situ* hybridization of the *A. burtoni* pituitary supports all pituitary galanin in this species being neural-derived (Fig. 3d).

Neuroanatomical locations of *GAL* cells in the *A. burtoni* brain were determined according to brain atlases from Burmeister et al. [95] and Cerdá-Reverter et al. [96]. The most anterior cell population identified was in the anterior preoptic area (POA) (Fig. 3c). Along the dorsal-ventral axis, this population spanned from the anterior commissure and approached the ventral edge of the brain. This group of cells displayed the most intense signal of all populations, as well as the greatest diversity in cell soma diameter (10–30 μm). Moving posteriorly, a small, sparse set of cells was present in the nucleus of the lateral tuberalis (NLT) (Fig. 3d). A few, faintly-stained cells were also observed along the midline, in the periventricular nucleus of the posterior tuberculum (Fig. 3e). In the caudal hypothalamus, *GAL*-expressing cells were distributed in the anterior portion of the nucleus of the lateral recess (NRL), and directly dorsal to the nucleus of the posterior recess (NRP) (Fig. 3f). The most posterior group was found in the hindbrain, bordering the vagal lobe (Fig. 3g). Hindbrain galanin cells have been previously described in the locus coeruleus of cyprinodonts [97].

The presence of galanin-expressing cells in the POA and NLT is conserved across ray-finned fishes. In contrast, the presence and locations of more posterior populations exhibits greater diversity across ray-finned fishes. The NRL and NRP are considered components of the fish homolog to the nonmammalian vertebrate paraventricular organ, which contains galanin-immunoreactive cells in amphibians [98, 99]. An NRL population has been described in *C. auratus* [100], and both NRP and NRL populations described in *Anguilla anguilla* (eel) [101], *Apteronotus leptorhynchus* (brown ghost knifefish) [102], and *O. mykiss* [103]. Only the POA and NLT populations were identified in the non-haplochromine cichlid *Alcolapia grahami* (Lake Magadi tilapia) [97]. The lack of more caudal populations in *A. grahami* could be due to differences in specificity between techniques, diversification of the galanin system within cichlids, or a combination of the two.

### Subfunctionalization of POMC prohormones

**Fig. 4:**
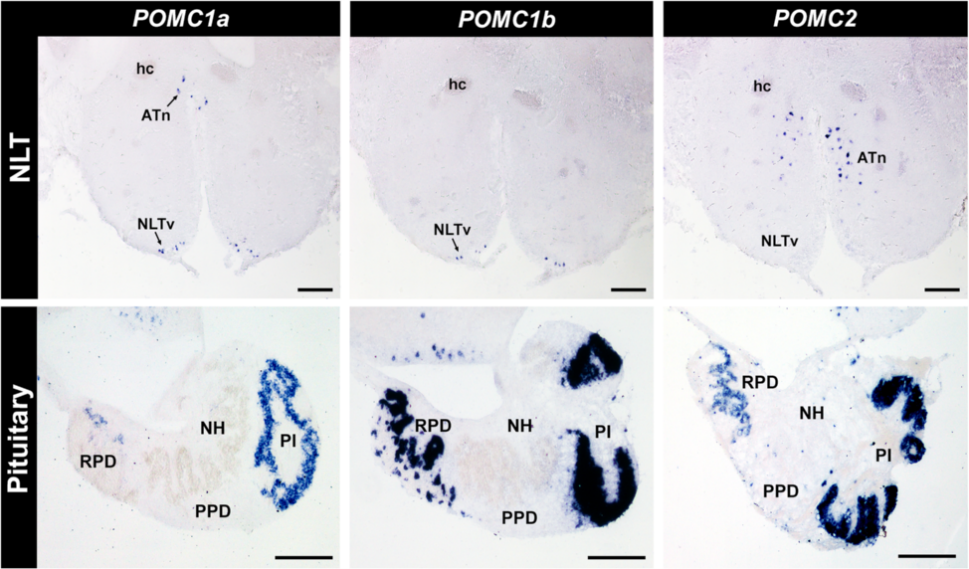
Distribution of *POMC1A*, *POMC1B*, and *POMC2* in the hypothalamus and pituitary gland of *Astatotilapia burtoni. In situ* hybridization shows *POMC1A* and *POMC1B* staining in NLTv and rostral ATn, and *POMC2* in ATn. All three *POMC*s were found in the RPD and PI of the pituitary. Brain is 20 μm transverse sections and pituitary is 20 μm sagittal sections. ATn, anterior tuberal nucleus; hc, horizontal commissure; NH, neurohypophysis; NLT, nucleus of the lateral tuberalis; NLTv, ventral part of NLT; PI, pars intermedia; PPD, proximal pars distalis; RPD, rostral pars distalis;. Scale bars = 100 μm

We show here the first exploration of three *POMC* prohormones in the same species on the cellular level in the brain and pituitary using *in situ* hybridization (Table 3). Expression of all three *POMC* prohormones was found in both the corticotrope (rostral pars distalis)- and melanotrope (pars intermedia)-containing regions of the *A. burtoni* pituitary (Fig. 4). *POMC1B* showed the most intense signal in the pituitary, particularly in the pars intermedia, which is consistent with reverse transcription polymerase chain reaction (RT-PCR) results [18]. Similarly, expression of three *POMC* prohormones of *V. moseri* was detected in whole pituitary samples [104].

**Table 3:** Localization of the proopiomelanocortin (*POMC*) gene family in the brain and pituitary of *Astatotilapia burton* *i*

Brain Region^a^	*POMC1A*	*POMC1B*	*POMC2*
Telencephalon			
Dm	-^b^	-	+
Dld	-	-	+
Dlg	-	-	+
Dlv	-	-	+
Diencephalon			
POA	-	-	+
NLT	+	+	+
ATn	-	-	+
Pituitary			
RPD	+	+	+
PI	+	+	+
Mesencephalon			
T	-	-	+
Metencephalon	-	-	-
Rhombencephalon			
NCC	-	-	+

^a^ Brain Region: *ATn* anterior tuberal nucleus, Dm, medial part of the dorsal telencephalon; Dld, Dlg, Dlv, dorsal, granular, and ventral zones of the lateral part of the dorsal telencephalon; *NCC* commissural nucleus of Cajal; *NLT* nucleus of the lateral tuberalis, *POA* preoptic area, *PI* pars intermedia, *RPD* rostral pars distalis, *T* tectum

^b^ +, detected; -, not detected

Localization of *POMC* prohormones within the brain showed that *POMC1A* and *POMC1B* occurred in the same locations, while *POMC2* was more widely expressed. *POMC1A* and *POMC1B* expression were restricted to two hypothalamic nuclei, the NLT and rostral anterior tuberal nucleus (ATn) (Fig. 4). There were numerous small (4–10 μm diameter) *POMC1A* and *POMC1B* cells in the ventral part of the NLT (NLTv) above the infundibular recess and pituitary, and a smaller population of larger neurons (10–15 μm diameter) located along the midline in the most rostral part of the ATn near the horizontal commissure. This expression pattern matches that of the single *POMC1* gene in *Tetraodon nigroviridis* (green spotted puffer) [105]. The range of *POMC2* expression in the *A. burtoni* brain and pituitary encompassed that of the *POMC1* genes but within the NLT, *POMC2* expression was more predominant in the medial and inferior parts of the NLT, while POMC1s were localized to the NLTv. *POMC2* expression also extended to the dorsolateral telencephalon, POA, tectum, and commissural nucleus of Cajal in the hindbrain (Additional file 5: Figure S1). This expression pattern is broader than that in *T. nigroviridis*, in which *POMC2* is restricted to the POA [105].

Although all three *POMC* genes are expressed in the hypothalamus and the pituitary, the tandem MS results suggest that *POMC1B* and *POMC2* peptide products are either not present, are at an undetectable level, or are expressed at different times than *POMC1A* in pituitary (Additional file 4: Table S3). Whether *POMC1B* and *POMC2*-derived peptides are present in other tissues or developmental stages remains to be explored. Increased brain *POMC1A* expression is associated with dominant status in adult *A. burtoni* [17], but it is unknown which brain regions contribute to this increase and whether *POMC2* exhibits similar social status-dependent expression.

It remains to be determined what function the extrahypothalamic *POMC*-expression serves, as well as whether *POMC1A* or *POMC2* are generally more varied in expression pattern across teleosts. For example, *β*-endorphin-like immunoreactive cells have been described in the thalamus and cerebellum of other teleosts [106, 107]. Since the beta-endorphin region of *POMC2* has degenerated in many teleost lineages, it is unclear which prohormones are involved.

## Conclusions

Our systematic survey of prohormone genes in the *A. burtoni* genome identified 167 sequences from 141 prohormone, 7 prohormone-related and 10 *PCSK* genes, with experimental evidence for numerous peptides derived from proteins encoded by many of these genes. In addition, tandem MS identified a possible novel fish-only neuropeptide from *VSTM2A*. Identification of peptides across fish species will facilitate functional testing of prohormone families, and whether there are any synergistic or collective mechanisms contributing to fish behavioral diversity. Two thirds of prohormone families contain duplicate genes, most deriving from the teleost WGD, indicating that this gene group has retained duplicates nearly three times the genome-wide average. These duplicates may serve as substrates for behavioral and physiological diversification within fishes and may have contributed to the remarkable speciation in the African cichlid species. In the case of *POMC*, we show that all three *A. burtoni POMC* genes are expressed in the hypothalamus and pituitary, but MALDI-TOF and tandem MS analysis of the pituitary suggest only one gene yields peptide products. Whether duplicates described in *A. burtoni* have undergone functional changes to give rise to different roles can be pursued in the wider context of the remarkably diverse African cichlids. The elucidation of *A. burtoni*’s prohormone complement comes at an exciting time in cichlid research, and follows recent developments in genome assembly and transgenic technologies.

## Methods

### Prohormone identification *in silico*

Detection of teleost prohormones requires a two-phase approach to address the impacts of WGD, tandem duplication, reciprocal gene loss, and ligand–receptor coevolution. In the first phase, orthologs of known prohormones and any paralogs are identified. Subsequently, the second phase searches for any previously unidentified prohormone paralogs across different genomes.

In the first phase, 109 candidate genes, including known gene duplications and possible pseudogenes, were derived from prior mammalian and avian studies [67, 108, 109]. Each sequence was searched in the genome using our previously documented approach [67]. The protein sequence of each candidate gene was matched to the *A. burtoni* genome assembly using TBLASTN with the default settings (*E*-value < 10 and BLOSUM62 scoring matrix) and filtering disabled on the cichlid data site (http://cichlid.umd.edu/cichlidlabs/kocherlab/bouillabase.html). All scaffold position matches with *E*-values < 1 were evaluated as possible prohormone genes to account for WGD, tandem duplication, and ligand–receptor coevolution. Partial matches were also used to query the *A. burtoni* EST database in order to provide a more accurate match as well as any alternative splicing. When there was no suitable BLAST match to a candidate gene, the other cichlid resources were used to confirm any missing candidate gene or provide a more suitable candidate. The resulting matches were classified into similar matches based on *E*-value and percentage identity to separate duplicated genes from genes from the same prohormone family. Prohormone protein sequences were predicted using the gene parsing tool Wise2 [110]. The final predictions were then bioinformatically screened for alignments to related genes in the same neuropeptide family across other species to ensure the accuracy of prediction.

Compared to tetrapods, each teleost prohormone was expected to have two paralogous copies due to the third tetraploidization. In the second phase, candidate genes with only a single match were further investigated for reciprocal gene loss. Initially the searches were conducted using the genomic resources of the other sequenced cichlids, primarily *O. niloticus*. Unsuccessful searches were then conducted in other published fish genomes, notably *Takifugu* species, *T. nigroviridis*, *O. latipes*, *Gasterosteus aculeatus* (three-spined stickleback), and *D. rerio*, to determine if a more closely related version of the candidate gene could be found. Finally, a literature search was conducted to find evidence that the candidate gene is duplicated in any fish species. Any potential sequence was further screened using the previous tools and databases to confirm the presence of a duplicated prohormone.

### Animals

Laboratory bred *A. burtoni* adults between 6 and 8.5 cm in standard length were housed in mixed sex communities in 60 l aquaria under conditions mimicking natural habitat conditions (26.5 °C; pH 8.5; 12 h dark: 12 h light with full spectrum illumination) [111]. Animals were fed daily with cichlid pellets and flakes (AquaDine, CA, USA). Animals were euthanized by rapid cervical transection prior to pituitary and/or brain dissection.

### MALDI-TOF MS of pituitary tissue

Freshly frozen individual pituitaries (three dominant males and three non-brooding females) were used for MALDI-TOF MS. Each pituitary was transferred onto a MALDI sample plate, divided into 10–15 pieces using electrolytically sharpened tungsten needles, each tissue piece was transferred onto a new sample spot and mixed with 0.5 μl of MALDI matrix (2,5-dihydroxybenzoic acid, 50 mg/ml of 50 % acetone). Spectra were manually acquired on a Bruker ultrafleXtreme mass spectrometer equipped with a smartbeam-II™ laser (Bruker Daltonics, MD, USA) operated at 1 kHz speed in reflectron mode. External calibration was performed using Bruker Peptide Mix II standards in identical matrix.

### Pituitary peptide extraction

Pituitaries were rapidly dissected from a second cohort of 5 adult animals (1 male, 4 females) and homogenized in 0.25 M acetic acid (Sigma-Aldrich, CA, USA) using a Dounce homogenizer. Homogenates were pooled and then centrifuged for 30 min at 4 °C and 15,000 x *g*. The supernatant pH was adjusted to ~4 using 1 M NaOH (Fisher, PA, USA) and desalted using Pierce C18 Spin Columns (Pierce, IL, USA) according to manufacturer’s instructions. Column eluate was then dried (Savant SpeedVac, Thermo Scientific, Waltham, MA, USA) and reconstituted in 0.1 % formic acid (Sigma-Aldrich).

### Pituitary peptide analysis by LC-MS/MS

Samples were first acidified and purified on stage tips and eluted in 60 % acetonitrile/40 % H_2_O, fractions were then dried (SpeedVac, Thermo Scientific) and reconstituted in 2 % acetonitrile/97.8 % H_2_O/0.2 % formic acid and injected onto a self-packed 15 cm C18 analytical column with a flow rate of 300 nL/min directly infused into the mass spectrometer. Ultra performance liquid chromatography (UPLC) was performed using a Waters Acquity system (Waters, Milford, MA). The electrospray ionization (ESI) ion trap (IT) mass spectrometer (LTQ Orbitrap Velos; Thermo Scientific) was set in data-dependent mode to fragment the top 8 most intense, multiply charged ions using higher-energy collisional dissociation (HCD). The survey scan mass resolution was set to 60 K and the HCD fragment ion resolution to 7.5 K.

### Bioinformatic peptide identification from the tandem MS data

We performed the peptide identification on the tandem MS data exported as an mzXML file using PEAKS Studio software versions 5.3 and 7.0 (Bioinformatics Solutions, Waterloo, Canada). The PEAKS workflow included creation of *de novo* sequence tags that were then queried against a database of predicted *A. burtoni* prohormones and a database of NCBI-predicted proteins from the AstBur1.0 assembly using both standard (PEAKS DB) and homology (SPIDER) searches [112]. Standard search identified the peptides whose sequences matched those in a database, while homology search revealed peptides with slightly different sequences, which could be due to polymorphism or database error. Search parameters included 20 ppm mass error tolerance for monoisotopic precursor ions and 0.1 Da for fragment ions, precursor charge state 1–5, no enzyme cleavage, and a maximum of three variable modifications (pyroglutamate from E and Q, acetylation of N-terminus or lysine, disulfide bond, oxidation and amidation). The search results were filtered with -10lgP of 20; all tandem MS spectra with scores lower than 30 were manually inspected and false positives removed.

### Tissue preparation for *in situ* hybridization

Brains and pituitaries from 4 adult *A. burtoni* (2 males, 2 females) were prepared for *GAL in situ* hybridization and brains and pituitaries from an additional group of 5 adults (2 males, 3 females) for all *POMCs*. These animals were separate from those used for MS experiments. Tissues were fixed in 4 % paraformaldehyde overnight at 4 °C, rinsed in PBS, and then cryoprotected with 30 % sucrose overnight at 4 °C. Tissues were then embedded in Tissue Tek OCT media (Sakura Finetek, MA, USA) in vinyl specimen molds (Sakura Finetek) and frozen on dry ice. Tissues were sectioned at 20 micron thickness. All *GAL* tissues were sectioned in the transverse plane. *POMC* tissues were sectioned in either the transverse (1 male, 2 female) or sagittal plane (1 male, 1 female). Sections were thaw-mounted onto three replicate slide sets (Superfrost White, VWR, PA, USA) and dried at room temperature for two nights. Slides were stored at –80 °C until processed for *in situ* hybridization.

### *In situ* hybridization

To localize *GAL*, *POMC1A*, *POMC1B*, and *POMC2*-expressing cells in brain and pituitary tissue, chromogenic *in situ* hybridization of brain and pituitary tissue was performed as previously described [51]. RT-PCR was used to amplify target sequences from *A. burtoni* whole brain cDNA and introduce T3 RNA promoter sequences to the template. For antisense probe generation, the T3 RNA promoter sequence was introduced in the reverse primer. For sense probe generation, the T3 RNA promoter sequence was introduced in the forward primer. We identified a target region of low sequence identity (~40 %) between the 3′UTRs of *POMC1A* ([GenBank : KC464872.1]) and *POMC1B* (Broad *A. burtoni* brain transcriptome [6], comp114_c0_seq1_indC_brain) mRNAs by sequence alignment using ClustalW (Geneious 8.0.5, Biomatters Inc.). The following primers were used to generate a template for cRNA antisense probe synthesis: *GAL* forward primer 5′-CTA GAT GGA CTA CAT GGA CAC AC-3′, *GAL* reverse primer 5′-AAT TAA CCC TCA CTA AAC GGA TTG GCC AGT-3′; *POMC1A* forward primer 5′- GAG AAA AGA GGG AGG GAT GGA G-3′, *POMC1A* reverse primer 5′-TGC AGT TGT GAA TA-3′; *POMC1B* forward primer 5′-AGA CGA GAA GAA GAT GAG GCA-3′, *POMC1B* reverse primer 5′-GTC TAA TTG CCT TG-3′; *POMC2* forward primer 5′-GAC CTC TTA CTC AGC GTT ATT C-3′, *POMC2* reverse primer 5′-AGA TAG CAA CGA GTT TGT GTA A-3′.

## Additional files

Additional file 1: Table S1. Location and accession numbers of identified *A. burtoni* prohormone and related genes. Genome scaffold locations and identities of supporting transcriptome data for all predicted *A. burtoni* prohormones and protein convertases. (CSV 27 kb) Additional file 2: Text S1. Predicted *A. burtoni* prohormone and related sequences in FASTA format. All *A. burtoni* prohormone and protein convertase sequences and names as predicted by *in silico* search. (FA 44 kb) Additional file 3: Table S2. Pituitary MALDI-TOF peptides confirmed by tandem MS. Sequences and masses peptides detected by both MALDI-TOF and tandem MS. (XLSX 11 kb) Additional file 4: Table S3.
*A. burtoni* pituitary peptides identified from tandem MS. Sequences and protein assignments of pituitary peptides detected by tandem MS. (CSV 87 kb) Additional file 5: Figure S1.
*POMC2* expression throughout the *A. burtoni* brain. Representative *in situ* hybridization images of *POMC2*-expressing regions in the *A. burtoni* brain. (PDF 275 kb)
