# Supplementary material for: Identification of prohormones and pituitary neuropeptides in the African cichlid, Astatotilapia burtoni
Source: BMC Genomics. 2016 Aug 19;17:660. doi: 10.1186/s12864-016-2914-9 (PMC4992253; doi:10.1186/s12864-016-2914-9)
Supplement: Additional file 5: Figure S1. — POMC2 expression throughout the A. burtoni brain. Representative in situ hybridization images of POMC2-expressing regions in the A. burtoni brain. (PDF 275 kb) [file 12864_2016_2914_MOESM5_ESM.pdf]

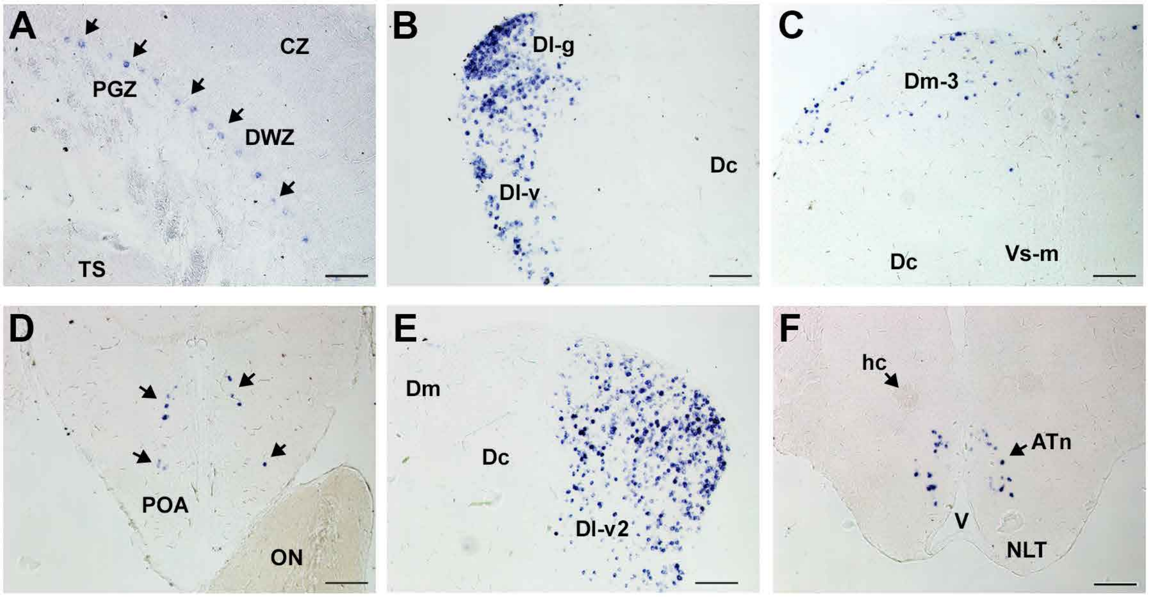

Additional file 5. Localization of *POMC2* expression throughout the *Astatotilapia burtoni* brain. *In situ* hybridization detected *POMC2*-expressing cells in (A) the tectum, (B-E) telencephalon, and (F) the hypothalamus. ATn, anterior tuberal nucleus; CZ, central zone; Dc, central part of the dorsal telencephalon; Dl-g, granular division of the lateral part of the dorsal telencephalon; Dl-v, ventral division of the lateral part of the dorsal telencephalon; Dl-v2, ventral subdivision of the lateral part of the dorsal telencephalon 2; Dm, medial division of the dorsal telencephalon; Dm-3, medial division of the dorsal telencephalon subdivision 3; DWZ, deep white zone; hc, horizontal commissure; NLT, lateral tuberal nucleus; ON, optic nerve; POA, preoptic area; PGZ, periventricular gray zone of the tectum; TS, torus semicircularis; Vs-m, medial division of the ventral telencephalon.
